# Impact of Combined Pollution of Ciprofloxacin and Copper on the Diversity of Archaeal Communities and Antibiotic-Resistance Genes

**DOI:** 10.3390/antibiotics13080734

**Published:** 2024-08-05

**Authors:** Meijuan Chen, Weiying Li, Haibo Teng, Wenxin Hu, Zhiqiang Dong, Dawei Zhang, Tianyi Liu, Quan Zheng

**Affiliations:** 1 College of Environmental Science and Engineering, Tongji University, Shanghai 200092, China; chenmeijuan2024@gmail.com (M.C.); 1710040@tongji.edu.cn (Z.D.); zhangdawei@tongji.edu.cn (D.Z.); 2 East Line Smart Water of China South-to-North Water Diversion Corporation Limited, Beijing 100071, China; tenghaibo2024@gmail.com (H.T.); huwenxin2024@gmail.com (W.H.); liutianyi2024@gmail.com (T.L.); rollins9190@gmail.com (Q.Z.)

**Keywords:** ciprofloxacin, archaea, antibiotic-resistance genes, stress and post effect

## Abstract

This study aimed to explore the response of archaeal communities and antibiotic-resistance genes (ARGs) to ciprofloxacin (CIP, 0.05–40 mg/L) and copper (Cu, 3 mg/L) combined pollution during stress- and post-effect periods in an activated sludge system. With the increase in the CIP concentration, the diversity of archaea decreased, but the richness increased under the stress of 10 mg/L CIP. Under stress and post effects, the change in unknown archaeal community structure was more significant than that of the known archaea. The relative abundance of unknown archaea was significantly reduced with the increase in CIP concentration. Meanwhile, there were certain archaea that belonged to abundant and rare taxa with different resistance and recovery characteristics. Among them, *Methanosaeta* (49.15–83.66%), *Methanoculleus* (0.11–0.45%), and *Nitrososphaera* (0.03–0.36%) were the typical resistant archaea to combined pollution. And the resistance of the abundant taxa to combined pollution was significantly higher than that of the rare taxa. Symbiotic and competitive relationships were observed between the known and the unknown archaea. The interactions of abundant known taxa were mainly symbiotic relationships. While the rare unknown taxa were mainly competitive relationships in the post-effect period. Rare archaea showed an important ecological niche under the stress-effect. Some archaea displayed positive correlation with ARGs and played important roles as potential hosts of ARGs during stress- and post-periods. *Methanospirillum*, *Methanosphaerula*, *Nitrososphaera* and some rare unknown archaea also significantly co-occurred with a large number of ARGs. Overall, this study points out the importance of interactions among known and unknown archaeal communities and ARGs in a wastewater treatment system under the stress of antibiotics and heavy metal combined pollution.

## 1. Introduction

It is well known that microorganisms and their metabolic activities are vital in maintaining the health of the global ecosystem, playing important roles in the circulation of nutrients, the degradation of pollutants, and the promotion of production [1]. However, only a limited number of microorganisms have been cultured, and most of the microbial species are still uncharacterized [2,3]. These unidentified microorganisms also play an important role [1], for which there is still scanty research. Antibiotics are widely applied in various contexts (animal husbandry, livestock breeding, and human medicine) and are difficult to be degraded and absorbed by organisms [4]. The enrichment of antibiotics in natural environments will flow into wastewater treatment plants (WWTPs), including quinolones, macrolides, sulfonamide, and tetracyclines, ultimately affecting the performance of WWTPs’ systems [5]. Among them, ciprofloxacin (CIP) is a quinolone commonly found in WWTPs, and the concentration of CIP was described as being relatively high in influent water within the range of 90.1–3327 ng/L [4,6]. Meanwhile, with the acceleration of industrialization, heavy metals are abundant in the influent and effluent of WWTPs, especially copper (Cu(II), 1–5 mg/L), with a high risk of threatening human health [7]. Therefore, the necessity of studying the antibiotics and heavy metals combined pollution in WWTPs has appeared. Meanwhile, antibiotics can induce the proliferation and spread of antibiotic-resistance genes (ARGs) [8]. Ulteriorly, ARGs have been shown to increase the abundance of antibiotic-resistant microorganisms with harm to human health [9]. In addition, some studies confirmed that the presence of heavy metals can also increase the risk of ARGs’ transmission [10,11]. Meanwhile, the activated sludge process is widely used in WWTPs for biological denitrification. And the microbial community is the decisive factor for the purification efficiency and stability of the system. Therefore, it is necessary to study the impacts of antibiotics and heavy metals combined pollution on activated sludge systems.

Activated sludge is composed of complex microbial communities, including eukaryotes, bacteria, archaea, and viruses. Most of the previous studies have focused on the effects of antibiotics and heavy metals on bacteria. High concentrations of azithromycin (40 mg/L) inhibited the relative abundance of ammonia-oxidizing bacteria (AOB) and promoted the enrichment of aerobic denitrification bacteria. AOB and some denitrifying bacteria obviously decreased in relative abundance under long-term exposure of 10 and 30 mg/L Cu [7]. And it has been confirmed that different bacteria had a specific response to different antibiotics, and some bacteria were potential hosts of ARGs [5]. Some potential pathogens (*Burkholderia*) and nonpathogenic bacteria (*Meiothermus* and *Sphingopyxis*) were identified as the possible hosts of ARGs [12]. Meanwhile, archaea also played an important role in the activated sludge system, e.g., ammonia-oxidizing archaea (AOA) [13] and methanogens [14]. In addition, some previous indicated that AOA could tolerate antibiotics pressure (CIP and terramycin) and resist cometabolic biodegradation [15,16]. A previous study displayed that some archaea were potential hosts of resistance genes, and the horizontal gene transfer (HGT) between archaea provided a pathway for the spread of resistance genes [17]. These results suggested the important role of archaea in ARGs’ transmission. These previous studies mainly investigated the impacts of antibiotics, heavy metals, and their combination on abundant and known archaea in their stress-effect period. However, the influences on rare or unknown archaea, comprising a majority of archaea on the planet [3,18], and the post effect of combined pollution are still unclear and need to be further explored.

According to the above arguments, the stress- and post effects of antibiotics and heavy metals combined pollution on archaea (known, unknown, abundant, and rare) and ARGs were explored. We assume that the stress response of known and unknown archaea to composite pollution is different, and both can serve as potential hosts for ARGs. In this research, CIP and Cu(II) were selected as the research objects. The main aims were (1) to investigate the impacts of combined pollution on known and unknown archaea; (2) to explore interactions among known and unknown archaea during stress- and post-effect periods; and (3) to evaluate the fate of ARGs and their archaeal potential hosts. This study could provide a practical reference for the ecotoxic effects of the combined pollution on archaea in activated sludge systems.

## 2. Result and Discussion

### 2.1. Variations of Archaeal Community Structures

**Figure 1:**
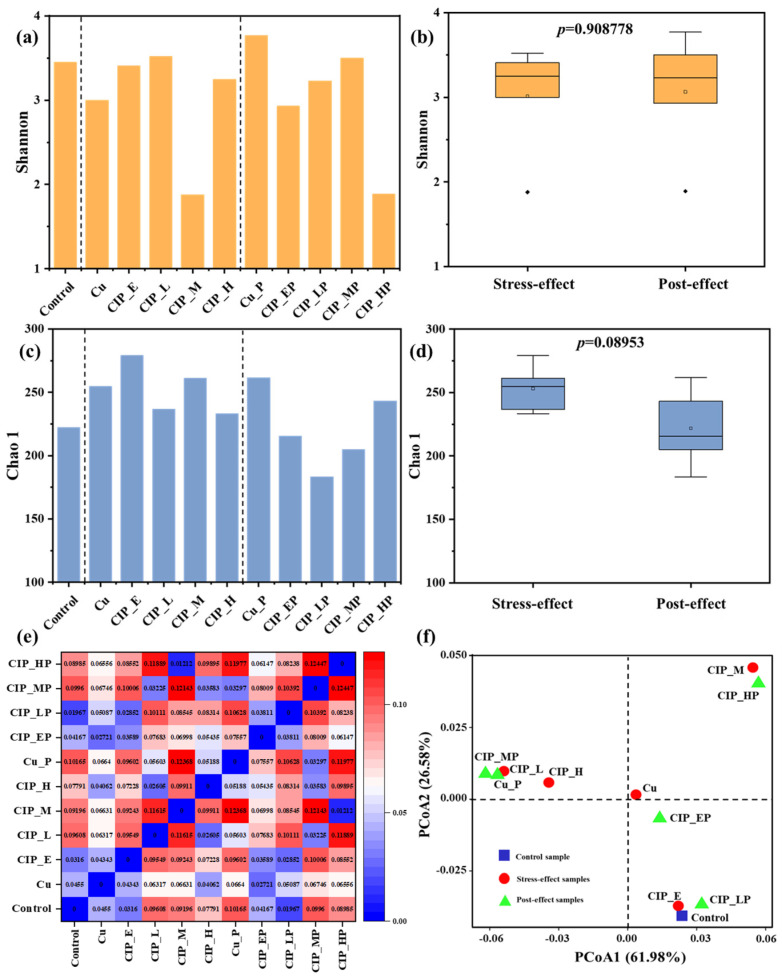
Variations in archaeal community diversities under combined pollution in stress- and post-effect samples. (**a**–**d**) changes and variance analysis of Shannon and Chao1 indexes; (**e**,**f**) the beta diversity and PCoA analysis of all archaea. Samples in control group and only with Cu group were named as Control and Cu, respectively. The samples in the stress-effect period under different concentrations of CIP and 3 mg/L of Cu were named as CIP_E (environmental concentration, E: 0.05 mg/L), CIP_L (low concentration, L: 1 mg/L), CIP_M (medium concentration, M: 10 mg/L), and CIP_H (high concentration, H: 40 mg/L). The corresponding samples in the post-effect period were Cu_P, CIP_EP, CIP_LP, CIP_MP, and CIP_HP. The rhombus in the (**b**) represented outliers.

The Good’s coverage value of all samples achieved 100%, which indicated that the archaeal communities were well represented. The alpha diversity analysis suggested that the variations of Shannon and Chao1 were slightly changed in stress- and post-effect samples (Figure 1a,c), except for the Shannon index in CIP_M and CIP_HP, indicating that the inhibitory effects of combined pollution on the diversity of microbial communities only occurred under high concentration pressure. Also, there were slight differences in Shannon and Chao 1 during the stress- and post-effect period (Figure 1b,d), suggesting that the effects of combined pollution on archaeal communities were noncontinuous, which was different from the effects on bacterial diversity and richness [16]. As shown in Figure 1e, the beta diversity in Cu and CIP_E were close to the Control, while there were significant difference with the increase in CIP concentration in other stress-effect samples, which verified that a higher concentration of CIP altered the archaeal community. Also, a similar phenomenon occurred in the post-effect samples. As shown in Figure 1f, the PCoA plot revealed the clusters the in stress- and post-effect samples, including Group1 (Control, CIP_E, and CIP_LP), Group2 (Cu and CIP_EP), Group3 (CIP_L, CIP_H, Cu_P, and CIP_MP), Group4 (CIP_M and CIP_HP), which indicated that the different concentrations of combined pollution resulted in significant evolution of the archaeal community structures. Interestingly, the effects of environmental concentration combined pollution were more significant than the pollution of Cu(II), which could be explained by the previous result that the formation of Cu(II) and CIP complexes reduced the concentration of the pollutants [19].

### 2.2. The Significant Fraction of Unknown Taxa in the Microbial Community

**Figure 2:**
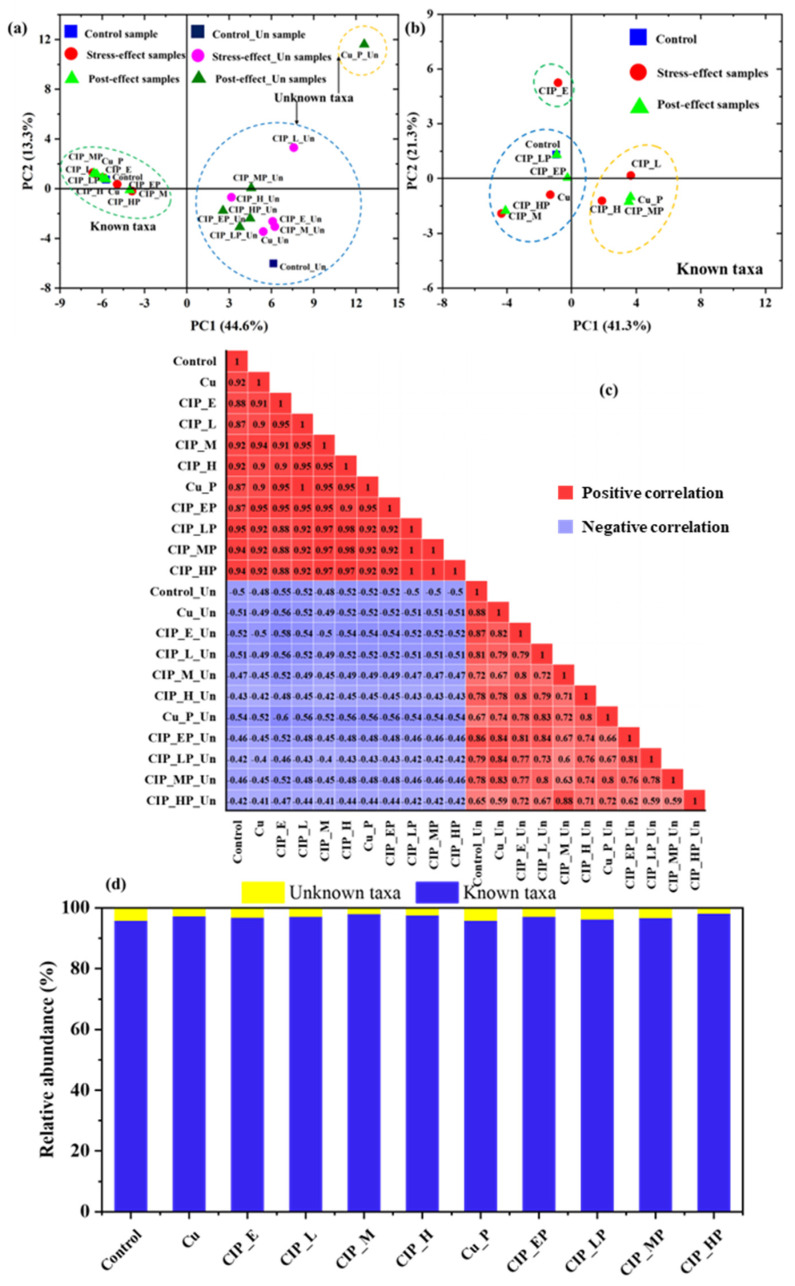
The responses of know–unknown archaeal community structures under CIP and Cu(II) combined pollution during stress- and post-effect periods. (**a**,**b**) PCA analysis of all known and unknown taxa; (**c**) the correlation analysis between known taxa and unknown taxa; (**d**) the changes in relative abundance in known taxa and unknown taxa. Samples in control group and only with Cu group were named as Control and Cu, respectively. The samples in the stress-effect period under different concentrations of CIP and 3 mg/L of Cu were named as CIP_E (environmental concentration, E: 0.05 mg/L), CIP_L (low concentration, L: 1 mg/L), CIP_M (medium concentration, M: 10 mg/L), and CIP_H (high concentration, H: 40 mg/L). The corresponding samples in the post-effect period were Cu_P, CIP_EP, CIP_LP, CIP_MP, and CIP_HP.

The previous study confirmed that “microbial dark matter” were top hubs in all environments and played an important role in their communities [3]. Thus, the archaea communities were divided into known and unknown taxa. The structures of all archaea community and known taxa were analyzed using PCA. For all archaeal communities (Figure 2a), known taxa and unknown taxa were distinctly distinguished, suggesting that the effects on archaeal community structures were differences between known and unknown taxa. Meanwhile, the samples of unknown taxa were more significantly far away from each other than known samples, indicating the influence of different pollutant concentrations on archaea of unknown taxa, especially for the contamination of Cu(II) alone. As for the responses of known taxa (Figure 2b), CIP_E, Group Ι (Control, Cu, CIP_M, CIP_EP, CIP_LP, and CIP_HP), and Group II (CIP_L, CIP_H, Cu_P, and CIP_MP) were divided. The stress- and post-effect samples with different concentrations of CIP were distributed in different groups, which might be due to the combined action between different concentration levels and experimental periods on archaea community structure, which were consistent with the bacterial community [16,20]. Meanwhile, Figure 2c shows the significant differences between known taxa and unknown taxa, indicating that the unknown archaea were weakly correlated with known archaea. However, the inner associations in known taxa were more significant than unknown taxa during stress- and post-effect periods, especially CIP_MP and CIP_HP (*p* = 1), which suggested that the archaea of known taxa were tolerant of CIP stress with different concentrations, while the unknown archaea were sensitive. As shown in Figure 2d, the relative abundances of archaea genera were stabilized, including 96~98% known genera and 2~4% unknown taxa, which indicated that known and unknown genera were tolerant to different concentrations of combined pollution. Overall, the responses of unknown archaeal genera to combined pollution were obviously different from known archaeal genera, which needs to be further studied.

### 2.3. The Response of Archaea Genera Based on Full-Scale Classification

**Figure 3:**
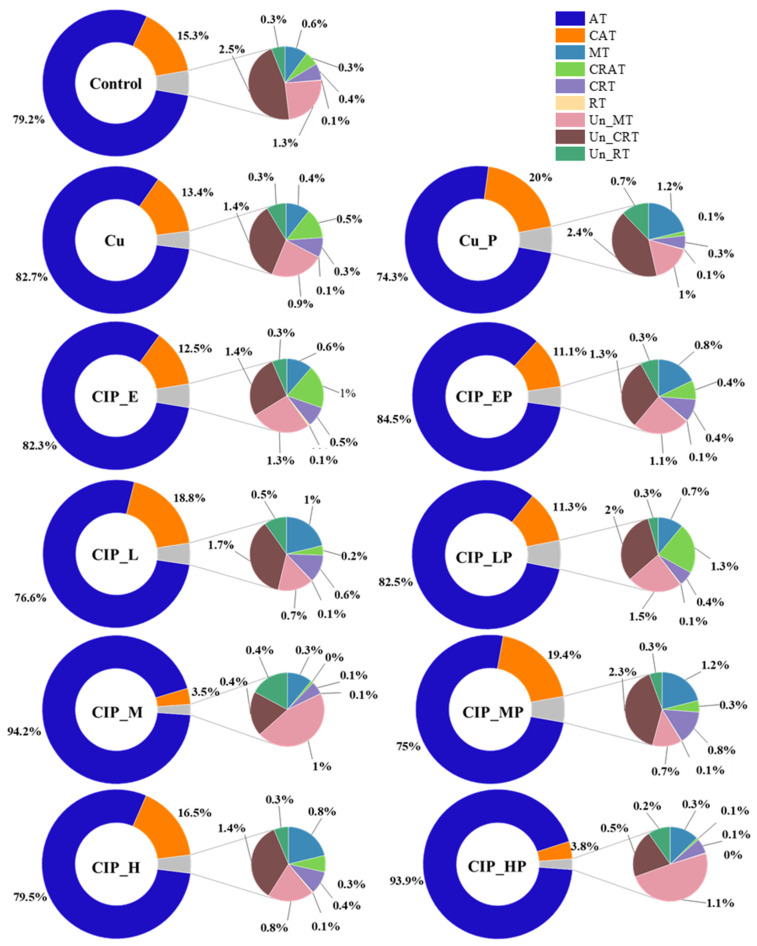
Effects of CIP and Cu combined pollution on known and unknown archaeal community compositions based on the full-scale classification method in stress- and post-effect samples. Samples in the control group and only with Cu group were named as Control and Cu, respectively. The samples in the stress-effect period under different concentrations of CIP and 3 mg/L of Cu were named as CIP_E (environmental concentration, E: 0.05 mg/L), CIP_L (low concentration, L: 1 mg/L), CIP_M (medium concentration, M: 10 mg/L), and CIP_H (high concentration, H: 40 mg/L). The corresponding samples in the post-effect period were Cu_P, CIP_EP, CIP_LP, CIP_MP, and CIP_HP.

According to a previous study, full-scale classification was used to deeply explore the response of archaeal genera to combined pollution [18]. In Known taxa, AT and CAT were the keystone taxa in the archaea community in all samples (Figure 3). The relative abundances of AT were close to Control in all stress- and post-effect samples, except CIP_M and CIP_HP, indicating the tolerance of dominant archaea to combined pollution. On the contrary, the relative abundances of CAT decreased in CIP_M and CIP_HP, which suggested that some archaeal genera of CAT were sensitive to higher concentrations and found it hard to recover. As for MT, CRAT, CRT, and RT, their relative abundances were stabilized during stress- and post-effect periods, which might be explained by the archaeal communities being resistant to the influences of a volatile environment [21]. Compared with known genera, the relative abundances of unknown taxa were more versatile (Figure 3). The relative abundances of Un_MT and Un_CRT decreased with the increase in concentrations of CIP and were increased in the post-effect period, suggesting that some unknown genera of Un_MT and Un_CRT were sensitive to combined pollution and easily recovered. Meanwhile, the relative abundances of Un_RT in all stress- and post-effect samples were still close to Control, which indicated some Un_RT may be resistant to combined pollution. These results confirmed the important role of unknown genera [3]. In order to further research, the known genera were divided into abundant taxa (AT, CAT, MT, and CRAT) and rare taxa (CRT and RT).

#### 2.3.1. Abundant Taxa

**Figure 4:**
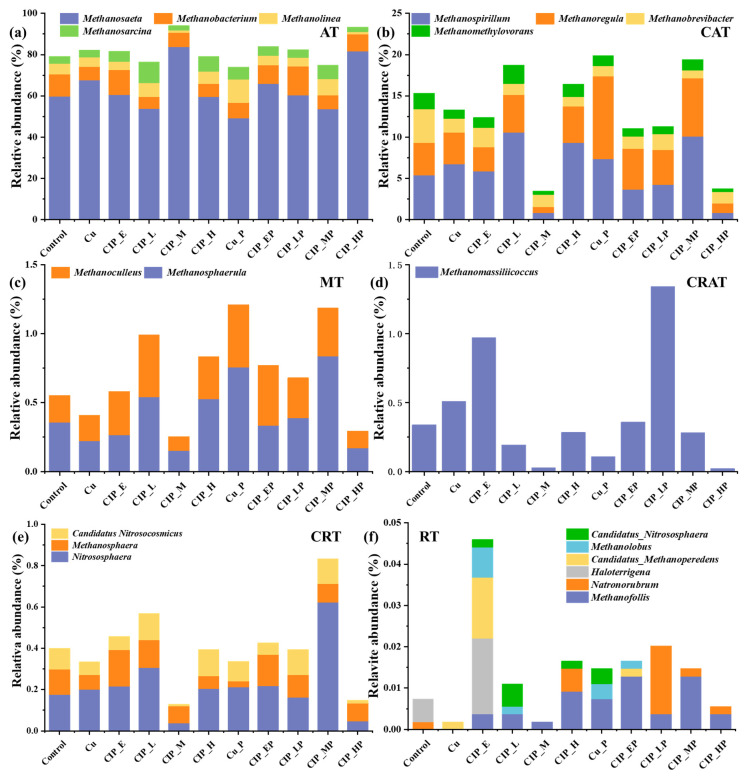
Variations in known archaeal community compositions of six taxa under combined pollution during the stress- and post-effect period. (**a**–**f**) the relative abundance of abundant taxa (AT), conditional abundant taxa (CAT), moderate taxa (MT), conditional rare or abundant taxa (CRAT), conditional rare taxa (CRT), and rare taxa (RT). Samples in the control group and only with Cu group were named as Control and Cu, respectively. The samples in the stress-effect period under different concentrations of CIP and 3 mg/L of Cu were named as CIP_E (environmental concentration, E: 0.05 mg/L), CIP_L (low concentration, L: 1 mg/L), CIP_M (medium concentration, M: 10 mg/L), and CIP_H (high concentration, H: 40 mg/L). The corresponding samples in the post-effect period were Cu_P, CIP_EP, CIP_LP, CIP_MP, and CIP_HP.

In this study, a total of 11 archaeal genera were classified as abundant taxa (Figure 4a–d), and all of them were functional methanogenic archaea, indicating that archaea might play an important role in methanogenesis [22]. Among them, *Methanosaeta*, *Methanobacterium*, *Methanolinea*, and *Methanosarcina*, which were the most dominant methanogenic genera with various operating conditions [23,24], were contained in AT (Figure 4a). *Methanosaeta* (49.15–83.66%) was the dominant genus in all samples. Its relative abundance in Cu, CIP_E, CIP_L, and CIP_H was close to Control and obviously increased in CIP_M, indicating that this genus was resistant to a certain degree of antibiotic pollution, which is supported by a previous study [24]. In post-effect samples, the relative abundance significantly decreased in Cu_P and CIP_MP, while it obviously increased in CIP_HP. Compared with Control, the relative abundances of *Methanobacterium* decreased in all stress-effect samples (except CIP_E), and then slightly increased in the post-effect period, suggesting that *Methanobacterium* were sensitive to Cu(II) and CIP pollution. The relative abundance of *Methanolinea* decreased in CIP_M and CIP_HP, while its relative abundance increased in Cu_P. Meanwhile, the relative abundance of *Methanosarcina* was only increased in CIP_L, and the relative abundances in other samples were close to Control. These results indicated the tolerance of dominant archaea to combined pollution.

As for CAT (Figure 4b), *Methanospirillum* (0.79–10.59%), *Methanoregula* (0.74–10.03%), *Methanobrevibacter* (0.92–4.09%), and *Methanomethylovorans* (0.36–2.26%) were detected. The relative abundance of *Methanospirillum*, known as the hydrogen-utilizing methanogens [25], increased in samples with a low concentration of contaminants and obviously decreased in CIP_M, suggesting that *Methanospirillum* was sensitive to CIP in higher concentrations. Its relative abundance decreased in some post-effect samples, especially CIP_HP, while significantly increasing in CIP_MP, which indicated the short-term post effects of combined pollution on this genus. Meanwhile, *Methanoregula* and *Methanomethylovorans* showed similar trends. The relative abundance of *Methanobrevibacter* was inhibited in all stress- and post-effect samples, which might be due to its sensitivity to Cu(II) and CIP pressure.

*Methanosphaerula* (0.15–0.83%) and *Methanoculleus* (0.11–0.45%) were classified in MT (Figure 4c). The total relative abundance of these genera decreased in Cu and CIP_M, compared with Control, and increased in other stress-effect samples, suggesting their obviously sensitivity to Cu(II) stress and slight resistance to the pressure of CIP. Moreover, their relative abundances increased in all post-effect samples, except CIP_HP, which indicated the post effect of a high concentration of combined pollution. The proportion of *Methanosphaerula*, a dominant hydrogenotrophic methanogen, showed similar variations. However, the relative abundance of *Methanoculleus*, the suppliers of methane in submarine methane hydrate deposits [26], showed slight variation during stress- and post-effect periods, except in CIP_M and CIP_HP, suggesting the resistance to combined pollution with a certain CIP concentration.

As for CRAT (Figure 4d), *Methanomassiliicoccus* (0.03–1.34%) was the specific genus. Compared with Control, the enrichment in the relative abundance of this genus occurred in Cu and CIP_E, but a reduction occurred in other stress-effect samples (especially CIP_M), indicating that this genus was sensitive to CIP with rising concentrations in a short-term period, which contradicted the results of long-term antibiotic pressure [27]. Moreover, this proportion obviously increased in CIP_LP and CIP_MP during the post-effect period, suggesting the restorability of *Methanomassiliicoccus*.

#### 2.3.2. Rare Taxa

In this study, a total of nine genera were classified as rare taxa. Among them, three genera belonged to CRT (Figure 4e), including *Nitrososphaera* (0.03–0.36%), *Methanosphaera* (0.03–0.18%), and *Candidatus Nitrosocosmicus* (0.01–0.13%). Among them, *Nitrososphaera* and *Candidatus Nitrosocosmicus*, the dominant AOA [13], showed similar variations in most stress- and post-effect samples, except CIP_M and CIP_HP, suggesting that AOA had resistance to combined pollution, which is consistent with a previous study [16]. The relative abundance of *Methanosphaera*, a typical methanogen which can survive in extreme environments [28], decreased in Cu and CIP_H, while it increased in other stress-effect samples, indicating its sensitivity to Cu(II) and tolerance to CIP in a certain concentration range. Meanwhile, its relative abundance slightly increased during the post-effect period, except for Cu_P.

As for RT (Figure 4f), *Methanofollis* (0–0.013%), *Natronorubrum* (0–0.018%), *Haloterrigena* (0–0.018%), *Candidatus_Methanoperedens* (0–0.015%), *Methanolobus* (0–0.007%), and *Candidatus_Nitrososphaera* (0–0.005%) were detected. These genera were irregularly distributed and were not dominant genera in any of the samples, indicating the inhibitions of combined pollution to rare archaeal genera, which is similar to the influences on bacterial genera [16]. *Methanofollis* was not identified in Control and Cu. However, it appeared in CIP_E, and its relative abundance increased with rising CIP concentration, reflecting that *Methanofollis* was resistant to CIP. Similar trends occurred in the post-effect periods. *Natronorubrum* and *Haloterrigena* were the typical halophilic archaea, which were observed in Control and some post-effect samples, suggesting these genera tolerated contaminants. Meanwhile, other genera were not observed in Control but were identified in specific samples, including *Candidatus_Methanoperedens* in Cu and CIP_E, *Methanolobus* in CIP_E, CIP_L, Cu_P, and CIP_EP, and *Candidatus_Nitrososphaera* in CIP_E, CIP_L, CIP_H, and CIP_EP, which indicated the tolerance of these genera in specific environments. All in all, the influences of different concentrations of combined pollution on unknown archaeal genera were more obvious than the known genera.

### 2.4. Network Analysis of Known Archaea, Unknown Archaea, and ARGs

#### 2.4.1. Interactions of Archaeal Genera

**Figure 5:**
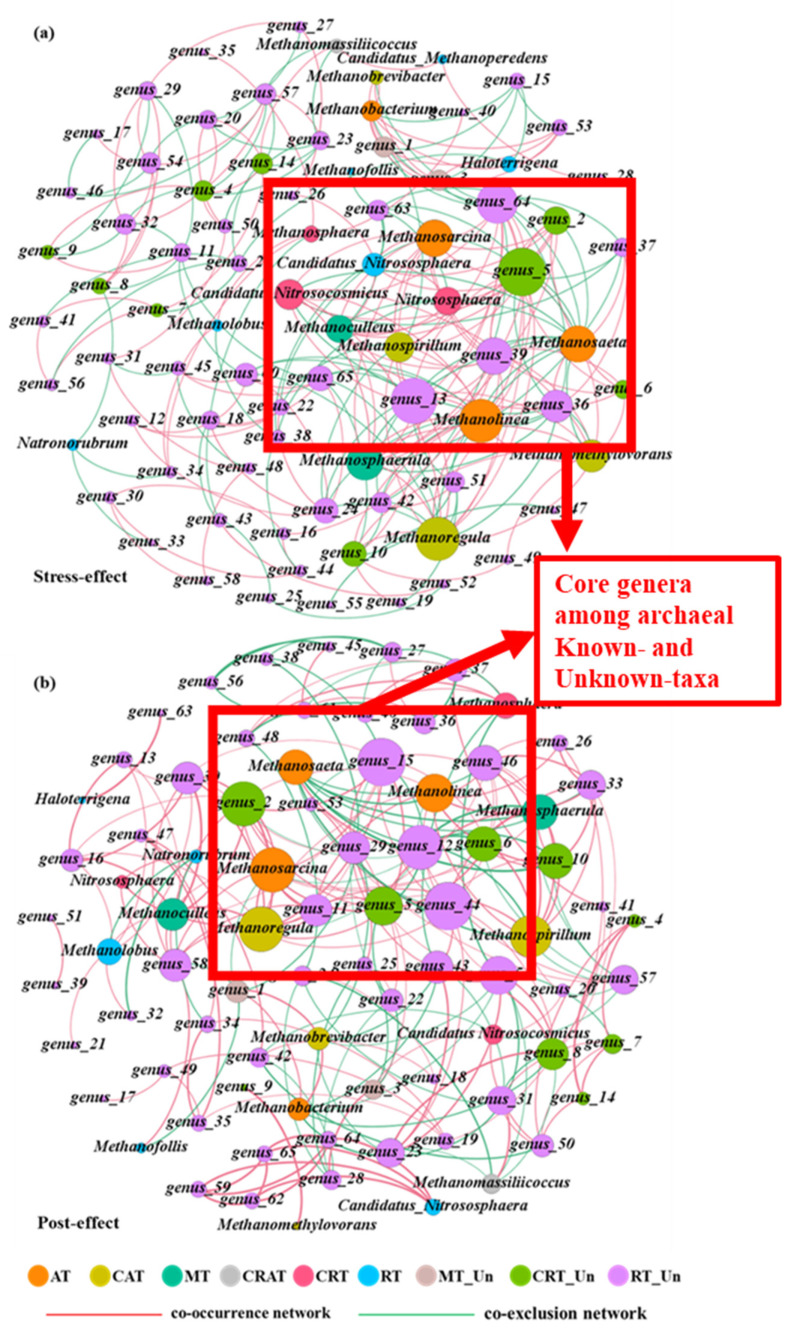
Co-occurrence or co-exclusion networks among known archaeal taxa (abundant taxa: AT, conditional abundant taxa: CAT, moderate taxa: MT, conditional rare or abundant taxa: CRAT, conditional rare taxa: CRT, and rare taxa: RT) and unknown archaeal taxa (unknown moderate taxa: Un_MT, unknown conditional rare taxa: Un_CRT, and unknown rare taxa: Un_RT) under CIP and Cu combined pollution during the stress-effect (**a**) and post-effect (**b**) period. Different colors of nodes represent the archaea among these taxa. Red and green lines represent positive and negative interactions with strong (Spearman r > 0.6 or r < −0.6) and significant (*p* < 0.05) correlations. The size of dots was proportional to the link numbers of each node. Core genera were highlighted by red boxes.

In this study, 255 and 238 edges were captured between 85 nodes in stress- and post-effect periods for the network (Figure 5), respectively. Among them, 59.21% of edges showed positive correlations, including inner associations of known (10.59%) and unknown taxa (25.49%) and their external associations (23.13%) during the stress-effect period (Figure 5a), indicating their co-occurrence relationships and the importance of unknown-taxa archaea in microbial networks, which supported the previous research [3]. For the inner associations of known-taxa genera, the co-occurrence patterns (27 edges) were common in the stress-effects period. The genera that belonged to CAT had the most positive relationship lines (14 edges), followed by AT (13 edges), MT (10 edges), and CRT (10 edges), suggesting that CAT was more closely related to other taxa, and abundant archaeal genera played important roles in the microbial interaction network. However, only one positive correlation edge occurred between CRAT and RT, which were significantly different from results of bacteria [5,12]. As for the unknown taxa, the co-occurrence pattern was slightly more than the mutual-exclusion pattern. RT_Un was the keystone taxa in their interactions, followed by CRT_Un and MT_Un, indicating the important roles of unknown and rare genera. Moreover, there were complex interactions among known and unknown taxa during the stress-effect periods. RT_Un and CRT_Un were the keystone taxa in their interactions, which showed positive correlation with known abundant genera (AT, CAT, CRAT, and MT) and negative correlation with known rare genera (CRT and RT), reflecting that the unknown rare genera were related to abundant genera and independent of rare genera. However, the MT_Un only had simple associations with known genera, including two, three, and one edges corresponding to AT, CAT, and RT, respectively, suggesting the unknown abundant genera were independent. In the post-effect period (Figure 5b), their co-occurrence patterns were more than in the stress-effect period among unknown taxa and the external associations, indicating that the post effects were obvious, which might suggest archaea could tolerate CIP pressure and easily recover [13]. Meanwhile, the positive interactions among known taxa were weakened and only occurred between some abundant genera during the post-effect period, which suggested the independence of rare genera. The main relationship between the inner known archaea and the known–unknown archaea was symbiosis during the stress- and post-effect periods. The core genera among known and unknown taxa were explored (Figure 5). The top five genera for network relationships were *genus_5* (belonged to CRT_Un), *genus_13* (belonged to RT_Un), *Methanolinea* (belonged to AT), *Methanoregula* (belonged to CAT), *genus_64* (belonged to RT_Un) during the stress-effect period and were changed to *genus_44* and *genus_15* (belonged to RT_Un), *Methanosarcina* (belonged to AT), *Methanoregula* (belonged to CAT), and *genus_2* (belonged to CRT_Un) in the post-effect period. Furthermore, *Methanoregula* and *Methanolinea* were important archaea owing to their interaction with unknown genera, while only *Methanosaeta* was negatively correlated with other known-taxa archaea in two periods. As for the unknown taxa’s inner associations, *genus_5* and *genus_44* was the core genus in the stress- and post-effect periods, respectively. Overall, the unknown genera obviously interacted with known taxa, and this needs further investigation.

#### 2.4.2. Potential Hosts of ARGs

**Figure 6:**
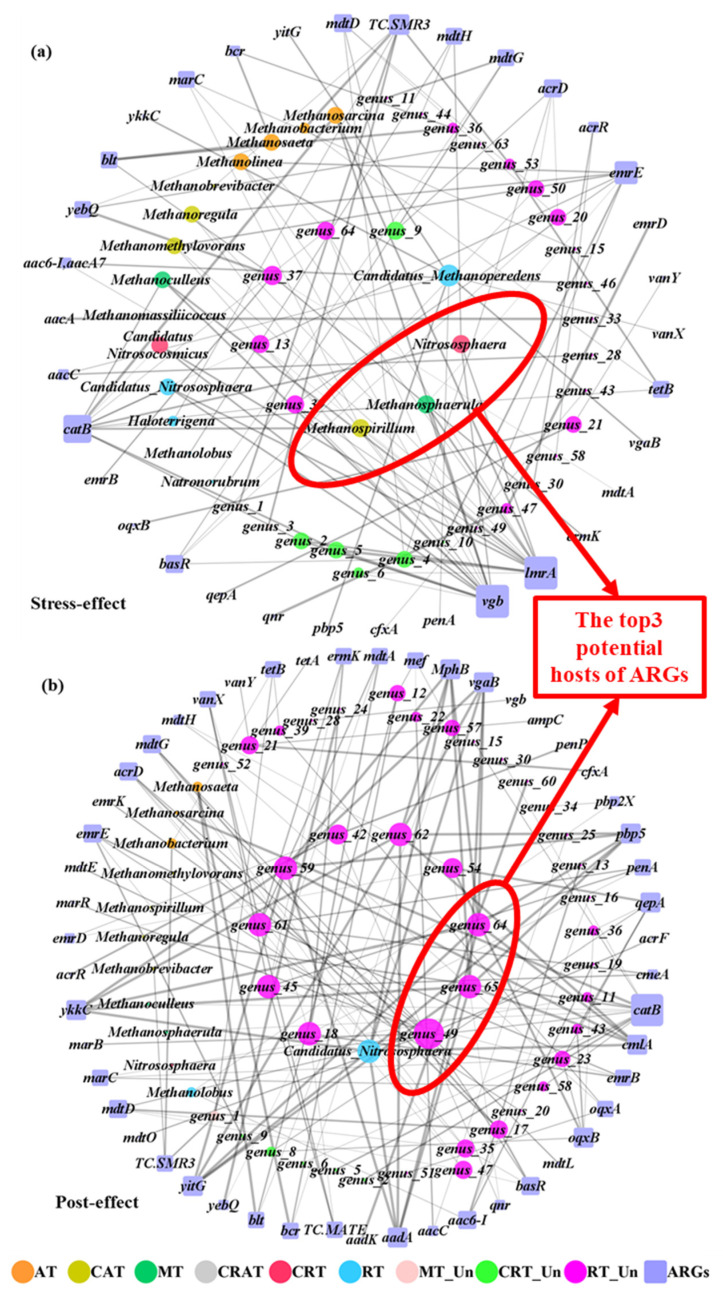
The potential hosts of ARGs in known archaeal taxa (abundant taxa: AT, conditional abundant taxa: CAT, moderate taxa: MT, conditional rare or abundant taxa: CRAT, conditional rare taxa: CRT, and rare taxa: RT) and unknown archaeal taxa (moderate taxa: Un_MT, unknown conditional rare taxa: Un_CRT, and unknown rare taxa: Un_RT) under combined pollution during the stress- (**a**) and post-effect (**b**) period. Different colors of dots represent the archaea among nine taxa. Purple squares represent the ARG subtype. The gray lines represent positive interactions with strong (Spearman r > 0.6 or r < −0.6) and significant (*p* < 0.05) correlations. The size of dots was proportional to the link numbers of each node. The top 3 potential hosts were highlighted with red circles.

As shown in Figure 6, the network analysis of positive relationships among ARGs and archaea showed the potential ARG hosts in the two periods (112 and 149 edges). There were complex correlations between ARGs and archaea in the stress-effect period, including CAT, MT, CRT, RT, CRT_Un, and RT_Un. Meanwhile, the core genera only belonged to RT and Un_RT in the post-effect period, which indicated the importance of unknown genera under post effects. Most of the rare genera were complex and unknown, which could lead to potential environmental risks if they carried a large number of resistant genes. In the stress-effect period, *Candidatus_Methanoperedens* in RT, which could utilize methane as the sole carbon source to degrade persistent organic contaminants [29], showed positive correlations with ARGs, including *aacC penA*, *pbp5*, *vgaB*, *vanX*, *ykkC*, and *yitG*. The genera of CAT (*Methanospirillum*), MT (*Methanosphaerula*), CRT (*Nitrososphaera*), CRT_Un (*genus_9*), and RT_Un (*genus_13*, *genus_37*, *genus_39*, and *genus_64*) also significantly co-occurred with a large number of ARGs, especially *emrE*, *lmrA*, *vgb*, *catB*, and *TC.SMR3*. *Candidatus_Nitrososphaera*, a moderately thermophilic AOA [30], co-occurred with ARGs in the post-effect period, suggesting the resistance of AOA to antibiotics, which supported the results of previous studies [13,16]. Meanwhile, a large number of unknown genera occupied an important position with respect to the occurrence of ARGs, especially *catB*, *oqxA*, *oqxB*, and *qepA*. Among them, *oqxA* and *oqxB* were the topical quinolone ARGs [13], which reflected the post effect of CIP. These results indicated that some unknown genera could be the hosts of ARGs. Overall, some of the unknown archaea also might be the potential hosts of ARGs, which should be allocated more attention.

Based on full-scale classification, this study identified the short-term stress response of known and unknown archaeal community compositions and interactions to CIP and Cu combined pollution. Moreover, the identification of different potential archaeal hosts of ARGs reveals the important role of archaeal communities in the transmission of ARGs. This study can provide a theoretical basis for evaluating the toxic effects of antibiotic and heavy metal combined pollution on the microbial community of activated sludge systems. However, there are still many aspects that can be further explored: (1) the effects and corresponding mechanisms of long-term stress of CIP and Cu combined pollution on the archaeal community and ARGs in activated sludge systems; (2) the stress mechanism of multiple typical antibiotics combined with Cu; (3) the intracellular and extracellular ARGs and their archaeal hosts; (4) the occurrence characteristics, co-occurrence patterns, and horizontal propagation mechanisms of ARGs based on quantitative analysis using high-throughput quantitative PCR; (5) the screening and identification of drug-resistant archaeal strains; and (6) the distribution characteristics and archaeal hosts of ARGs in system effluent under combined pollution stress.

## 3. Materials and Methods

### 3.1. Setup of Batch Experiments

The batch experiments were constructed with an environmental concentration of copper (3 mg/L) and different concentrations of CIP (0.05–40 mg/L) to explore the short-term impact of combined pollution on activated sludge. Copper sulfate and CIP were purchased from Shanghai MacLean Biochemical Co., Ltd. (Shanghai, China) and Energy Chemical (Hefei, Anhui, China) Co., Ltd., respectively. The concentrations of CIP were chosen according to previous studies [16,31,32,33], including four conditions (environmental:0.05 mg/L; low: 1 mg/L; medium: 10 mg/L; and high: 40 mg/L). The batch experiments without any contaminants and only containing copper (3 mg/L) were named as Control and Cu, respectively. Other batch experiments with both copper and environmental, low, medium, and high concentrations of CIP were named as CIP_E, CIP_L, CIP_M, and CIP_H, respectively.

All batch experiments were established in 500 mL conical flasks, and each batch experiment had 250 mL of working volume with a 50% drainage ratio. Synthetic wastewater was used in the experiments, which was chosen according to a previous study [34]. Activated sludge was collected from an aeration tank in a full-scale municipal WWTP in Beijing. The mixed-liquor suspended solids (MLSS) volume was 4500 mg/L in each batch experiment. Dissolved oxygen (DO), pH, and water temperature were controlled at 6–8 mg/L, 7.5–8.0, and 25–27 °C, respectively, which were continuously monitored with a multi-parameter water quality meter (WTW Multi3630 IDS, Xylem Analytics, San Diego, CA, USA). In order to explore the impact of CIP and copper combined pollution and keep the “in situ” condition, eight cycles were under exposure to dosed pollutant stress, and seven cycles were their post effects, which were named as the stress-effect period and post-effect period, respectively.

### 3.2. DNA Extraction and Archaeal Sequencing Analysis

At the end of the eighth and fifteenth cycles, three parallel activated sludge samples were obtained from each batch experiment and mixed. A total of eleven samples were collected including one sample in the control group (Control), five samples in the stress-effect group (Cu, CIP_E, CIP_L, CIP_M, and CIP_H), and others in the post-effect group (Cu_P, CIP_EP, CIP_LP, CIP_MP, and CIP_HP). Total genomic DNA were extracted with an E.Z.N.A.^®^ Soil DNA Kit (Omega Bio-tek, Norcross, GA, USA). All samples were stored at −20 °C.

In order to explore the responses of archaeal communities to combined pollution, the 16S rRNA genes (V3-V4 region) of archaea were applied with the universal primer set 314F (5′-CCTACGGGNGGCWGCG-3′) and 805R (5′-GACTACHVGGGTATCTAATCC-3′) [35] and sequenced with an Illumina Miseq PE300 sequencer (Illumina, San Diego, CA, USA) in Ovison Technology Co., LTD. (Beijing, China). The raw sequencing data of 16S rRNA genes were stored in a Sequence Read Archive (SRA) database with accession number PRJNA779200, and all raw sequencing data were organized using the Quantitative Insights Into Microbial Ecology (QIIME) pipeline package [36]. Then, high-quality sequences were obtained after removing the putative chimeras from raw sequencing data. These sequences were classified into an operational taxonomic unit (OTU) with >97% sequence similarity via the UPARSE algorithm. SILVA database release 132 (http://www.arb-silva.de/, accessed on 8 October 2021) was used to annotate different OTUs. The Chao1 and Shannon indexes were used to assess archaeal community diversity. And the archaeal community structures were analyzed via principal component analysis (PCA).

### 3.3. Full-Scale Classification and ARGs Analysis

All known archaeal communities (known taxa) were divided into six taxa using the full-scale classification method according to a previous study [18], with 1% and 0.1% of relative abundance as the thresholds, including abundant taxa (AT), conditional abundant taxa (CAT), moderate taxa (MT), conditional rare or abundant taxa (CRAT), conditional rare taxa (CRT), and rare taxa (RT). Meanwhile, the majority of archaeal taxa were uncharacterized [3]. A total of 65 unknown OTUs were detected in this study, which were named *genus_1*, *genus_2*, *genus_3*, etc. To explore the responses to combined pollution, this method was also adopted to classify unknown archaea into three taxa, with unknown moderate taxa (Un_MT), unknown conditional rare taxa (Un_CRT), and unknown rare taxa (Un_RT).

ARGs were analyzed using PICRUSt (phylogenetic investigation of communities by reconstruction of unobserved states), which was widely used in previous research [5,16,37]. ARGs were corresponded with the 104 Kyoto Encyclopedia of Genes and Genomes Orthologs (KOs) based on a previous study [38].

### 3.4. Statistical Analysis and Network Analysis

In this study, principal component analysis (PCA) was used to analyze the characteristics of archaeal community structure among different samples. The correlation matrix was constructed with Spearman’s rank correlation coefficients (SRCCs), and the correlation among known and unknown archaea, ARGs, and archaea were analyzed, with the indication of significant correlations (*p* ≤ 0.05). All significant correlations were further visualized using Gephi 0.9.2 (known and unknown archaea) or Cytoscape 3.8.0 (ARGs and archaea) to construct the network relationships.

## 4. Conclusions

The diversity decreased and the richness increased under the 10 mg/L CIP and 3 mg/L Cu combined pollution pressure. The known archaeal community structure was more stable than the unknown archaeal community during stress- and post-effect periods. With the increase in the CIP concentration, the relative abundances of the unknown archaeal communities were significantly reduced. *Methanosaeta*, *Methanoculleus*, and *Nitrososphaera* were the typical resistant archaea. The rare unknown archaea were the core genera of the stress- and post-effect periods. Some specific known abundant archaea and unknown rare archaea were the major potential hosts of ARGs.

## Data Availability

Data are contained within the article.
